# Therapeutic Effectiveness of Interferon-α2b against COVID-19 with Community-Acquired Pneumonia: The Ukrainian Experience

**DOI:** 10.3390/ijms24086887

**Published:** 2023-04-07

**Authors:** Aleksandr Kamyshnyi, Halyna Koval, Olha Kobevko, Mykhailo Buchynskyi, Valentyn Oksenych, Denis Kainov, Katerina Lyubomirskaya, Iryna Kamyshna, Geert Potters, Olena Moshynets

**Affiliations:** 1 Department of Microbiology, Virology, and Immunology, I. Horbachevsky Ternopil National Medical University, Majdan Voli 1, 46001 Ternopil, Ukraine; 2 Department of Clinical Immunology, Allergology and Endocrinology, Bukovinian State Medical University, Teatralnaya Square, 2, 58002 Chernivtsi, Ukraine; 3 Department of Infectious Disease, Chernivtsi Regional Clinical Hospital, Holovna, 137, 58000 Chernivtsi, Ukraine; 4 Department of Clinical and Molecular Medicine (IKOM), Norwegian University of Science and Technology, 7028 Trondheim, Norway; 5 Department of Obstetrics and Gynecology, Zaporizhzhia State Medical University, Maiakovskyi Avenue 26, 69000 Zaporizhzhia, Ukraine; 6 Department of Medical Rehabilitation, I. Horbachevsky Ternopil National Medical University, Majdan Voli 1, 46001 Ternopil, Ukraine; 7 Antwerp Maritime Academy, Noordkasteel Oost 6, 2030 Antwerp, Belgium; 8 Department of Bioscience Engineering, University of Antwerp, Groenenborgerlaan 171, 2020 Antwerp, Belgium; 9 Biofilm Study Group, Department of Cell Regulatory Mechanisms, Institute of Molecular Biology and Genetics, National Academy of Sciences of Ukraine, 150 Zabolotnoho Str., 03680 Kyiv, Ukraine

**Keywords:** IFN-α2b, COVID-19, therapeutic effectiveness, hospital stay, SpO2, CT-diagnosed lung injuries

## Abstract

Despite several targeted antiviral drugs against SARS-CoV-2 currently being available, the application of type I interferons (IFNs) still deserves attention as an alternative antiviral strategy. This study aimed to assess the therapeutic effectiveness of IFN-α in hospitalized patients with COVID-19-associated pneumonia. The prospective cohort study included 130 adult patients with coronavirus disease (COVID-19). A dose of 80,000 IU of IFN-α2b was administered daily intranasally for 10 days. Adding IFN-α2b to standard therapy reduces the length of the hospital stay by 3 days (*p* < 0.001). The level of CT-diagnosed lung injuries was reduced from 35% to 15% (*p* = 0.011) and CT injuries decreased from 50% to 15% (*p* = 0.017) by discharge. In the group of patients receiving IFN-α2b, the SpO2 index before and after treatment increased from 94 (92–96, Q_1_–Q_3_) to 96 (96–98, Q_1_–Q_3_) (*p* < 0.001), while the percentage of patients with normal saturation increased (from 33.9% to 74.6%, *p* < 0.05), but the level of SpO2 decreased in the low (from 52.5% to 16.9%) and very low (from 13.6% to 8.5%) categories. The addition of IFN-α2b to standard therapy has a positive effect on the course of severe COVID-19.

## 1. Introduction

Severe acute respiratory syndrome coronavirus 2 (SARS-CoV-2) first appeared in 2019 and caused the coronavirus disease 2019 (COVID-19), which resulted in a global pandemic [1]. This pandemic has triggered severe social and economic distress around the world and the largest global recession since the Great Depression [2,3].

The first COVID-19 cases in Ukraine were registered on 3 March 2020. Within half a year and despite a strict lockdown, four epidemic waves had appeared in Ukraine with approximately 5 million cases confirmed by the Center for Systems Science and Engineering (CSSE) at Johns Hopkins University (https://index.minfin.com.ua/reference/coronavirus/ukraine/, accessed on 24 July 2022).

Since there were initially no specific antiviral drugs or vaccines to control SARS-CoV-2, a wide variety of symptomatic treatment strategies have been proposed, including potential antiviral compounds such as ribavirin [4], lopinavir/ritonavir [5], remdesivir [6], nelfinavir [7], Arbidol [8] and chloroquine [6]. Convalescent plasma and protective monoclonal antibodies were also recommended therapies [9,10].

SARS-CoV-2-specific monoclonal antibodies and convalescent plasma represented another effective treatment strategy to protect at-risk patients, such as immunocompromised and elderly patients, from a severe or lethal course of infection [11]. However, the treatment effectiveness may vary depending on the predominant variant. As it was recently shown, the anti-spike monoclonal antibodies maintained a similar efficacy when the Alpha and Beta variants predominated; however, they showed a reduced effectiveness during the Delta variant epoch [12].

However, even though there are currently several effective vaccines and targeted antiviral drugs against SARS-CoV-2, such as molnupiravir and Paxlovid [13], the application of type I interferons (IFNs) still deserves the attention of virologists as an alternative antiviral strategy.

IFNs are pleotropic cytokines with antiviral activity which have already been reported to be effective in a number of respiratory and non-respiratory viral infections [14,15,16,17], as well as against different variants of COVID-19 [18,19,20]. The announced effectiveness of IFN-α in the treatment of patients with severe COVID-19 has been ambiguous: some work indicated a lack of positive effects [21,22], while at the same time, the effectiveness of such a treatment was confirmed in cohort studies [23,24] as well as clinical trials [25,26]. Our work aimed to assess the clinical effectiveness of IFN-α in hospitalized patients with COVID-19.

## 2. Results

**Figure 1:**
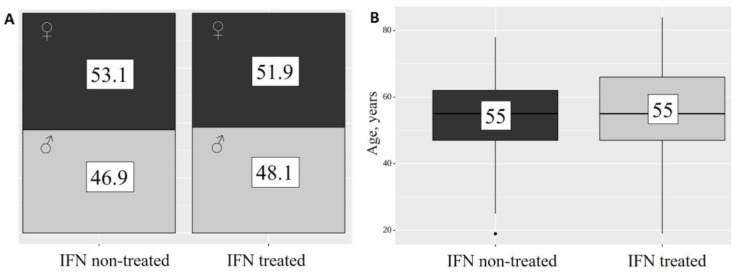
Patient characteristics. Distribution of sex (**A**) and age (**B**) within the non-treated (dark grey) and treated (light gray) patient groups.

As presented in Table 1, out of the 130 people who were in the hospital with COVID-19, 81 (62.3%) received IFN-α2b treatment (Figure 1).

**Figure 2:**
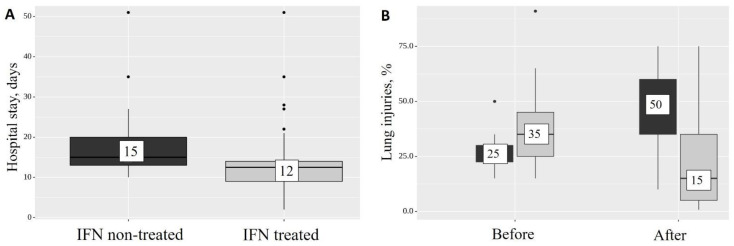
Treatment results. Length of hospital stay in control and treatment group (**A**), and analysis of lung injuries observed by CT (**B**) before and after the treatment period with (light gray) and without (dark gray) IFN-α2b treatment.

The addition of IFN-α2b to the standard therapy reduced the length of hospital stay from 15 to 12 days (*p* < 0.001) (Figure 2A), and reduced CT-diagnosed lung injuries at the time of discharge from 35% to 15% (*p* = 0.011) (Table 2 and Figure 2B). At the same time, compared with the group of patients who did not receive IFN-α2b, the percentage of CT injuries in treated patients by the end of their hospital stay decreased from 50 to 15% (*p* = 0.017) (Figure 2B).

**Figure 3:**
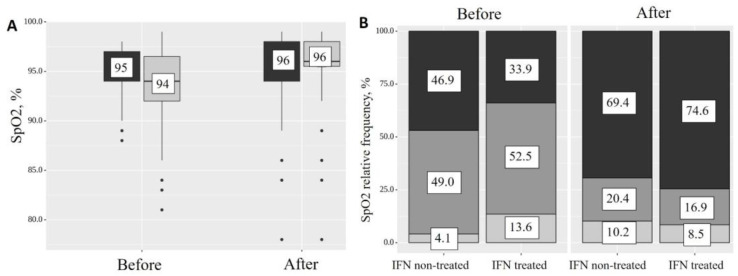
SpO2 dynamics before and after control (dark gray) and IFN-α2b treatment (light gray) (**A**) and SpO2 relative frequency before and after control and IFN-α2b treatment (**B**).

Initially, the level of SpO2 in the IFN-α2b-treated group was lower than in the non-treated group (*p* = 0.031) (Figure 3A); however, upon discharge from the clinic, there was no significant difference between the group which received the treatment and the group which did not (*p* = 0.553) (Figure 3A). However, in the group of patients receiving IFN-α2b, the SpO2 index before and after treatment increased from 94 (92–96, Q_1_–Q_3_) to 96 (96–98, Q_1_–Q_3_) (*p* < 0.001). The results were more intriguing when we categorized SpO2 scores into categorical groups (normal saturation—96–100%; low—90–95%; and very low—below 90%) (Figure 3B and Table 3). After IFN-α2b therapy, the percentage of patients with normal saturation increased (from 33.9% to 74.6, *p* < 0.05), but the level of SpO2 decreased in the low (from 52.5% to 16.9%) and very low (from 13.6% to 8.5%) categories (Figure 3B and Table 3).

**Figure 4:**
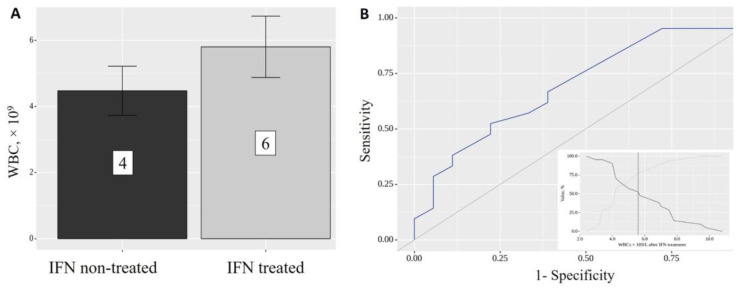
White blood cells (WBCs) following IFN-α2b treatment (**A**); ROC curve of WBC elevation prediction following INF treatment (**B**).

IFN-α2b treatment significantly increased the level of leukocytes from 4 × 10^9^/L to 6 × 10^9^/L (*p* = 0.028, Figure 4), but not that of any other blood cell types.

The area under the ROC curve was 0.701 ± 0.084 with a 95% CI: 0.537–0.865 (Figure 4B). The resulting model was statistically significant (*p* = 0.031). The cut-off value of WBCs after IFN-α2b treatment corresponding to the highest Youden’s J statistic was 5.600 × 10^9^/L. If the WBC value after IFN-α2b treatment was greater than or equal to this value, IFN-α2b treatment had a significant effect. The sensitivity and specificity of the method were 52.4% and 77.8%, respectively.

**Figure 5:**
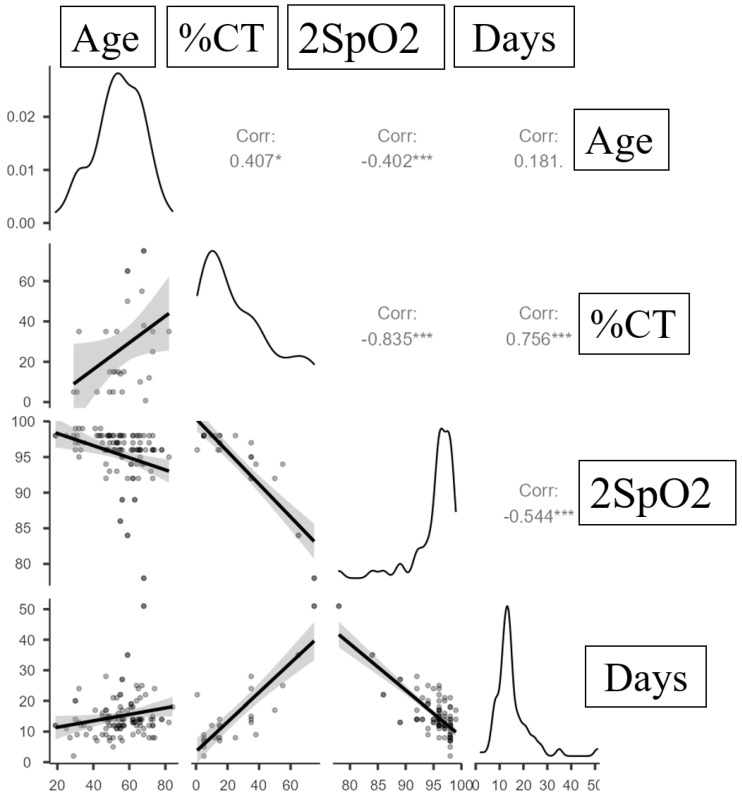
Regression line characterizing the dependence of days in hospital on %CT injuries, 2SpO2 and age. Statistical significance of correlations when * *p* < 0.05 and *** *p* < 0.005. Grey zones indicate 95% confidence intervals.

We performed a correlation analysis of the association between quantitative variables using Spearman’s correlation coefficient (Table 4 and Figure 5).

A strong positive association was inferred between the duration of the hospital stay (days in hospital) and the %CT injuries, which could be described by the linear regression equation: Y_Days in hospital_ = 0.481 × X_%CT injuries_ + 3.494. For a 1% increase in %CT injuries, an increase in the duration of the hospital stay of 0.481 days should be expected. According to the coefficient of determination (R^2^) of the resulting model, 72.9% of the observed variance in the number of hospital days was explained.

A close negative association between the number of days in the hospital and the 2SpO2 level was inferred from the linear regression equation: Y_Days in hospital_ = −1.518 × X_2SpO2_ + 160.056. With a decrease of 1% in the 2SpO2, an increase of 1.518 days in the hospital should be expected. The R^2^ of the resulting model indicates that 61.4% of the observed variance in the number of hospital days was explained.

A strong negative association between %CT injuries and 2SpO2 was estimated. The observed dependence of %CT injuries with 2SpO2 is described by the linear regression equation: Y_%CT injuries_ = −3.476 × X_2SpO2_ + 354.393. With a decrease of 1% in the 2SpO2 value, an increase of 3.476 CT injuries should be expected. The R^2^ of the resulting model indicates that 79.8% of the observed variance in the %CT injuries was explained.

**Figure 6:**
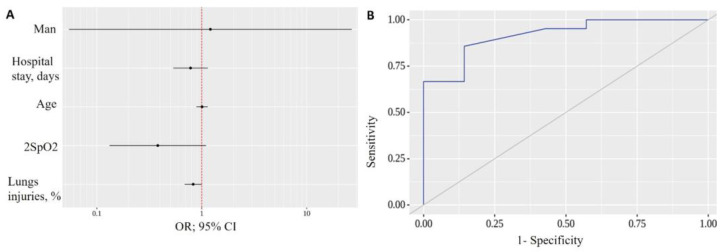
(**A**) Odds ratios estimate with corresponding 95% CIs for predictors included in the IFN-α2b treatment model; (**B**) ROC curve characterizing the dependence of the IFN-α2b treatment on value of logistic function P and analysis of the sensitivity and specificity of IFN-α2b treatment depending on the value *p* of the logistic function.

Subsequently, a predictive model was developed to estimate the therapeutic effects of IFN-α2b treatment conditioning on sex, age, days in hospital, % CT injuries and 2SpO2 using binary logistic regression based on 28 observations. The observed relation can be described by the following equations:

P = 1/(1 + e^−z^) × 100% (1)

z = 101.659 + 0.189X_men_ + 0.006X_Age_ − 0.246X_Days in hospital_ − 0.188X_% CT injuries_ − 0.965X_2SpO2_ (2)

where P indicates the probability of the therapeutic effect on a patient treated with IFN-α2b, X_men_ is the sex of the patient (0—women, 1—men), X_Age_ is the age of the patient, X_Days in hospital_ is the number of days the patient spent in the hospital, X_% CT injuries_ is the percentage of CT injuries and X_2SpO2_ is the 2SpO2 value. The resulting regression model is statistically significant (*p* = 0.013). Based on the value of Nagelkerke R^2^, the model explains 59.6% of the observed IFN-α2b treatment variance (Table 5 and Figure 6A).

When evaluating the dependence of the probability of the IFN-α2b treatment on the value of the logistic function P using the ROC analysis, the curve shown in Figure 6B was obtained. The area under the ROC curve was 0.918 ± 0.052 with a 95% CI: 0.816–1.000. The resulting model was statistically significant (*p* = 0.001). The cut-off value of the logistic function P which corresponded to the highest Youden’s J statistic was 0.702. If the logistic function P was greater than or equal to this value, IFN treatment took place. The sensitivity and specificity of the method were 85.7% and 85.7%, respectively (Figure 6B).

## 3. Discussion

IFNα treatment for COVID-19 has been reported on in more than 180 studies, as we recently analyzed [27]. Among them, there were 64 case reports/series, 54 retrospective/prospective cohort studies, 20 case–control studies, 15 clinical trials, 18 cross-sectional studies, 4 registry studies, 2 longitudinal studies and 1 multinational network cohort study. These studies were conducted in 14 countries (Argentina, Brazil, China, Cuba, France, India, Iran, Malaysia, Qatar, Russia, South Korea, Turkey, the United Arabs Emirates (UAE) and the United States of America (USA)). Most of the studies originated in China. There have been no studies conducted in Ukraine but the current one. The given therapeutic forms included solutions or suspensions, and the corresponding routes of administration (ROA) of IFNα treatment in these reports included inhalation or nebulization, subcutaneous injection, intramuscular injection, intravenous injection, a combination of inhalation and injection, injection without a reported site, nasal drops and spray, or the ROA was not reported.

IFNα inhalation later became a part of the standard treatment in China, hence why most studies reporting the use of IFNα inhalation came from China. Other studies using IFNα inhalation included Argentina, Qatar and Russia.

Inhalation/Nebulization or nasal spray was the most commonly reported ROA. The safety of IFNα inhalation was demonstrated in two studies, which reported no difference in the proportion of COVID-19 patients receiving IFNα treatment between those with and without delayed-phase thrombocytopenia, nor between survivors and non-survivors [28,29]. Furthermore, IFNα inhalation seemed to have beneficial effects on the liver during COVID-19 infection. One retrospective cohort study showed an association between IFNα inhalation and lower risks of elevated alanine aminotransferase (>40 U/L) in patients aged between 32 and 56 with (*n* = 86) and without non-alcoholic fatty liver disease (*n* = 194) [30]. These seemingly beneficial impacts of IFNα on the liver are somewhat intriguing, given that liver toxicity is a noted side effect of IFN-α2. Since IFN-α2 has been reported to be associated with several autoimmune diseases [31], and due to the absence of any empirical data on IFN-α2 intranasal application for hospitalized patients with COVID-19-associated pneumonia at the time of the current project, a recommended-by-manufacturer dose of IFN-α2 (80,000 IU daily) was used. Here, we should also note that the experimental increase in the dosage up to 600,000 IU daily did not show any toxic side effects but improved clinical outcomes with regard to COVID-19 (Moshynets O., personal observations).

**Figure 7:**
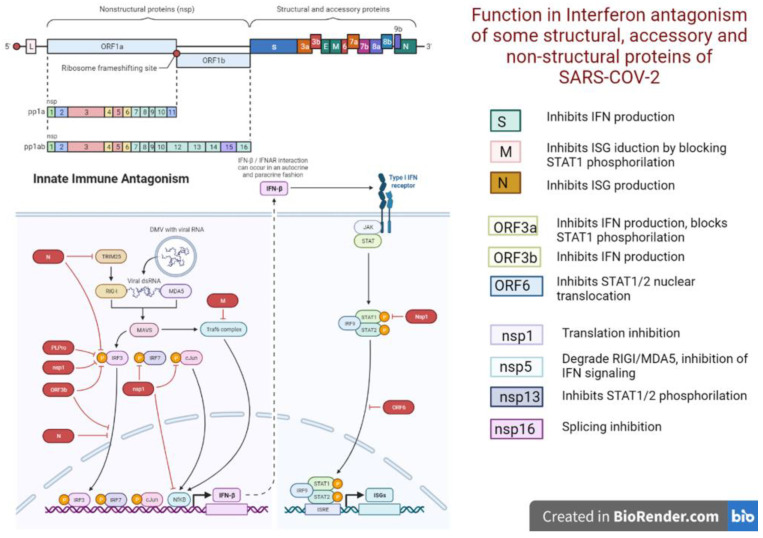
Ways SARS-CoV-2 proteins block interferon signaling.

Type I IFNs comprise a large family of molecules, including 13 members of the alpha family and 1 member of the beta family, and represent the oldest evolutionary system against viral infections, dating back over 450 million years [32]. Type I IFNs work in both autocrine and paracrine responses, inducing the expression of various interferon-stimulated genes (ISGs) that confer antiviral activity on host cells [33]. Many viral species, including SARS-CoV-2, have evolved various mechanisms to evade the antiviral function of type I IFNs. Up to 10 SARS-CoV-2 proteins have been identified to counteract the antiviral activity of IFNs [34] (Figure 7).

For instance, the non-structural protein 16 (NSP16) inhibits the splicing of mRNA and reduces the recognition of viral RNA by intracellular helical cases, NSP1 leads to a general inhibition of mRNA translation by binding to 18S ribosomal RNA in the mRNA input canal, and NSP8 and NSP9 disrupt protein traffic through the membrane. These three mechanisms independently result in a reduced production of type I IFNs by the affected cell [35]. Consequently, any IFN which was synthesized against these barriers and which had exited the cell may not have come in contact with its receptors because they are also partially blocked by ORF3a. Furthermore, any signal that eventually enters the target cell will be blocked at the level of formation of transcription factors IRF3, IRF7 or STAT1. Specifically, ORF6 inhibits STAT1 and STAT2 phosphorylation and STAT1 nuclear translocation, which blocks the transcription of interferon-stimulated genes and the formation of PKR and OAS [36].

A large number of clinical trials of type I IFNs have currently been registered on the clinicaltrials.gov website, in which the compound is being studied either alone (NCT04293887 and NCT04320238) or in combination with other drugs (NCT04254874, NCT04276688, NCT04273763, NCT04315948, NCT04350684 and NCT0435034381). For example, Zhou et al. (2020) [37] showed that treatment with IFN-α2b—in combination with umifenovir or not—significantly reduced the duration of the virus being detectable in the upper respiratory tract and shortened the duration of the elevated activity of inflammatory markers (IL-6 and CRP). Meng et al. (2020) [38] prospectively evaluated IFN-α1b nasal drops to prevent the infection of medical staff with SARS-CoV-2 and showed that IFN-α1b can serve as an effective prophylactic against COVID-19.

While the effects of prophylactic administration of IFNs at an early stage of the disease are obvious, the possibility of using type I IFNs in patients with severe COVID-19 has recently been questioned by some clinicians due to a common misconception about the possibility of an IFN-stimulated increase in inflammation and cytokine storms [39,40,41]. Despite early reports of suppressed IFN production, there is emerging evidence that patients with severe COVID-19 have a sustained type I IFN response that contrasts with the delayed, possibly suppressed, interferon response seen early on during infection [42]. A sustained type I IFN response may exacerbate hyperinflammation as COVID-19 progresses to a severe disease through a variety of mechanisms. This even led to the launch of a clinical trial on the use of monoclonal antibodies that deplete plasmacytoid dendritic cells, which could potentially lead to a decrease in the production of type I IFNs (https://clinicaltrials.gov/ct2/show/NCT04526912 (accessed on 14 February 2023)).

The emergence of these misunderstandings was mostly due to prevailing ideas about the purely pro-inflammatory effects of type I IFNs. However, the reality is that these same IFNs can also have immunomodulatory effects, reduce inflammation and cause cytokine storms. Thus, upon infection with the persistence-prone LCMV (lymphocytic choriomeningitis virus), type I IFNs upregulate the expression of programmed death-ligand 1 (PD-L1), and this upregulation leads to the depletion of CD8+ T cells and a decrease in immunopathology [43]. Type I IFNs, released during chronic infections, create an immunosuppressive environment by increasing the expression of PD-L1 and IL-10 via dendritic cells (DCs). These immunoregulatory DCs cause the depletion of CD4+ T cells. Interestingly, the effects of type I IFNs on the functional activity of T-regulatory cells are also ambiguous; however, several studies have shown their ability to enhance the suppressor potential of T-regulatory cells, including by increasing the production of the suppressor cytokine IL-10 [44]. The impact of type I IFN signaling on the activation and differentiation of CD4+ T-cell subpopulations remains poorly defined, and studies report conflicting evidence of a beneficial or detrimental role, depending on the context of the infection [45]. At the same time, interferons can support the expansion of Th1 and Tfh in the acute phase of infection, but cause their depletion during the chronic phase [46]. Type I IFNs can induce antigen-specific T cells to produce IL-10, which in turn, negatively regulates the Th17-mediated inflammatory and autoimmune response [47].

Some concerns regarding IFN therapy might include possible side effects. However, the Solidarity trial did not demonstrate any sign of a cytokine storm following IFN application [48]. In another randomized trial, Davoudi-Monfared et al. (2020) [49] evaluated interferon beta-1a in severe COVID-19 cases. The study group had a higher discharge rate at day 14 (66.7% vs. 43.6%) and a lower mortality rate after 28 days (19% vs. 44%, *p* = 0.015).

A recently published meta-analysis on selected types of immune therapies, including type I IFNs, found significantly lower chances of death (OR, 0.19; 95% CI, 0.04–0.85; *p* = 0.03) for recipients of this type of IFN [50]. A systematic review of the efficacy of IFN-α therapy was carried out by Nakhlband et al. (2021) [51] and Lu et al. (2022) [52]. The first group of researchers found that the time of viral clearance and PCR-negative (days) in most studies were decreased in the INF-α + standard care group. The mean length of time until the patient samples became virus negative in the INF-α-treated group and in the standard group were 27.3 and 32.43 days, respectively. Likewise, the average number of days of hospitalization was found to be lower in the INF-α group (18.55 vs. 24.36) [53]. Buchynskyi et al. [27] shows that IFN-α does not increase the survival of hospitalized COVID-19 patients but may increase the number of patients discharged from the hospital.

It should be noted that data on the nature of delayed IFN production are contradictory. Some early studies have shown that SARS-CoV-2 infection induces low levels of type I IFNs and type III IFNs with a moderate ISG response [53]. A more recent study examining peripheral blood from patients with varying degrees of severity of COVID-19 also found that type I IFN responses were severely impaired in patients with severe or critical COVID-19, as indicated by low levels of IFN-I and ISG, despite increased production of tumor necrosis factor (TNF) and IL-6 and increased inflammatory responses controlled by NF-kB [54]. However, there are conflicting results about increased ISG expression, and in a single-cell RNA sequencing study of peripheral blood mononuclear cells (PBMC) from hospitalized patients with COVID-19, various ISGs were activated in classical monocytes [55]. Interestingly, a comparison of single-cell RNA sequencing in patients with severe COVID-19 and patients with severe influenza showed that patients with COVID-19 had unique hyperinflammatory signatures for all types of immune cells, especially in the upregulation of inflammatory responses caused by TNF and IL-1, whereas type I IFN and type II IFN responses predominated in patients with severe influenza [56]. Type I IFN responses occurred simultaneously with inflammatory responses driven by TNFα and IL-1β in classical monocytes from patients with severe COVID-19, suggesting that type I IFNs may play an important role in exacerbation.

## 4. Materials and Methods

### 4.1. Study Design and Data Collection

The prospective cohort study included 130 adult patients with coronavirus disease (COVID-19) who were treated in the Infectious Diseases Department of the Chernivtsi Regional Clinical Hospital from November 2020 to May 2021. All patients were hospitalized for clinical indications of SARS-CoV-2 infection, which was confirmed by laboratory RT-PCR testing of a nasopharyngeal swab. Patients had been showing symptoms up to 7 days before hospitalization.

### 4.2. Clinical Characteristics of the Patients

As presented in Table 1 and Figure 1, the project included 130 patients with community-acquired COVID-19 pneumonia, which was confirmed by computed tomography (CT) of the chest. All patients underwent a diagnostic examination as well as monitoring in accordance with national recommendations. The patients had neither been vaccinated nor had COVID-19 disease before. The inclusion criteria were: (1) Patients diagnosed with COVID-19 by laboratory confirmation of SARS-CoV-2 via reverse transcriptase-polymerase chain reaction assays of the throat, which were assessed via swabs. (2) Severe or moderate COVID-19 infection meeting at least one of the following: (a) respiratory distress, defined as a respiratory rate ≥ 30 times/min (severe COVID-19) or ≥22 times/min (moderate COVID-19); (b) oxygen saturation ≤ 90% at rest (severe COVID-19), or in the range of 91–95% (moderate COVID-19); and (c) the symptoms showed progressive aggravation, and a chest X-ray or CT indicated that the lesions had progressed more than 50% within 24–48 h. (3) Patients with clear clinical outcomes (discharged). (4) Patients with community-acquired COVID-19 pneumonia, which was confirmed by computed tomography (CT) of the chest. The exclusion criteria were: (1) patients with incomplete medical records (e.g., transfer to other hospitals); (2) patients who were intubated, dead or discharged within 24 h of admission; (3) patients that were pregnant or had acute lethal organ injury (e.g., acute myocardial infarction, acute pulmonary embolism or acute stroke), acquired immune deficiency syndrome or leukemia; and (4) patients younger than 18 years. These specific criteria were established to avoid the non-uniform enrolment of patients, which could skew the interpretation of the results.

Moderate COVID-19 was defined by the following symptoms: fever above 38 °C; respiratory rate more than 22/min; shortness of breath during exercise; pneumonia (CT infiltrates in the lungs <45% of the pulmonary area); and SpO2 > 90%.

Severe COVID-19 was defined by the following symptoms: respiratory rate greater than 30/min; oxygen saturation (SpO2) ≤ 90%; the ratio of arterial oxygen partial pressure (PaO2 in mmHg) to fractional inspired oxygen, or PaO2/FiO2, ≤ 300 mm Hg; and progression of changes in the lungs typical of COVID-19 pneumonia (CT infiltrates in the lungs > 45–50% of the pulmonary area).

All patients were under standard treatment according to the national treatment protocol for COVID-19, which included: symptomatic antipyretic therapy (paracetamol or ibuprofen); anticoagulant therapy (low-molecular-weight heparins such as enoxaparin at a dose of 40 mg (4000 IU anti-Xa)); antimicrobial treatment of co-infections (amoxicillin/clavulanate plus macrolides (azithromycin or clarithromycin) or cephalosporins of the II–III generation plus macrolides (azithromycin or clarithromycin)); corticosteroids (0.15 mg/kg IV of dexamethasone once a day (maximum dose 6 mg) for 7–10 days); and non-invasive oxygen support. The duration of a hospital stay ranged from 7 to 51 days.

### 4.3. Randomization, IFN-α Treatment and Outcomes

The selected patients were randomly divided into treatment and control groups by simple open randomization. The treatment group consisted of 81 patients (62.3%). The control group included 49 patients (37.7%). The groups did not substantially differ in comorbidities. The treatment group of patients received nasal interferon alpha-2b (IFN-α2b) in addition to the standard therapy (described above).

Starting the first day of hospitalization, IFN-α2b was administered in the form of a nasal spray through 2 spray-doses into each nasal passage 4 times a day using 5 mL vials of Nasoferon (JSC “Farmak”, Kyiv, Ukraine) that consisted of 100,000 IU per ml and 5000 IU per spray-dose, which was a total of 80,000 IU per day for 10 days. The study protocol met the requirements for biomedical research and was approved by the Local Ethics Committee of the Chernivtsi Regional Clinical Hospital as protocol N10, dated 3 October 2020.

The primary outcome was the length of the hospital stay. The secondary outcomes included the level of CT-diagnosed lung injuries at the time of discharge and the after–before dynamics of SpO2 level due to IFN-α2b treatment.

### 4.4. Statistical Analysis

Continuous variables were described as median values (interquartile ranges (IQRs)) and compared with a Mann–Whitney test. Categorical variables were described as frequencies (percentages) and compared with a chi-squared test or Fisher’s exact test where appropriate. All inter-group comparisons were between the IFN-α2b group and the control group. The development of a prognostic model for the probability of a binary outcome was carried out using logistic regression. The Nagelkerke R^2^ was used as a measure of model performance.

Receiver operating characteristic (ROC) analysis was used to assess the diagnostic performance of quantitative variables in predicting a categorical outcome. The optimal cut-off value of the quantitative variable was estimated using Youden’s J statistic. A two-sided α of less than 0.05 was considered statistically significant.

## 5. Conclusions

1. Adding IFN-α2b to the standard therapy for patients with severe COVID-19 reduces the length of the hospital stay by 3 days (*p* < 0.001).
2. Treatment with IFN-α2b reduces the level of CT-diagnosed lung injuries from 35% to 15% (*p* = 0.011) at the time of discharge and, compared with the group of patients who did not receive IFN-α2b, the percentage of CT injuries decreases at the end of the hospital stay from 50 to 15% (*p* = 0.017).
3. In the group of patients receiving IFN-α2b, the SpO2 index before and after treatment increased from 94 (92–96, Q_1_–Q_3_) to 96 (96–98, Q_1_–Q_3_) (*p* < 0.001), while the percentage of patients with normal saturation increased (from 33.9% to 74.6, *p* < 0.05), but the level of SpO2 decreased in the low (from 52.5% to 16.9%) and very low (from 13.6% to 8.5%) categories.
4. Considering the contradictory results obtained regarding the strength of the response to type I IFNs in patients with severe COVID-19, more accurate information is required for the appropriate therapeutic use of type I IFNs.

## Figures and Tables

**Table 1:** Descriptive statistics for quantitative and categorical variables.

**Quantitative Variables**	**M ± SD/Me**	**95% CI/Q_1_–Q_3_**	**n**	**min**	**max**
Age, M ± SD	54 ± 14	52–56	130	19	84
Days in hospital, Me	14	12–17	117	7	51
Initial CT% injuries, Me	35	25–45	83	5	91
Final CT% injuries, Me	15	9–36	28	1	75
Initial SpO2, Me	95	93–97	116	81	99
Final SpO2, Me	96	95–98	108	78	99
**Categorical Variables**	**Categories**		**Abs.**		**%**
Sex	women		68		52.3
	men		62		47.7
IFN-α2b treatment	IFN non-treated		49		37.7
	IFN treated		81		62.3
Initial SpO2	normal		48		41.4
	low		56		48.3
	very low		12		10.3
Final SpO2	normal		78		72.2
	low		20		18.5
	very low		10		9.3

M—mean, Me—margin of error, SD—standard deviation, CI—confidence interval, Qn—quartile, min—minimum, max—maximum.

**Table 2:** Analysis of the level of % CT damage before and after IFN-α2b treatment.

IFN Treatment	Follow-Up Periods				*p* Initial vs. Final
	%CT Injuries 1 (Initial)		%CT Injuries 2 (Final)		
	Me	Q_1_–Q_3_	Me	Q_1_–Q_3_	
IFN-α2b non-treated	25 (*n* = 7)	22–30	50 (*n* = 7)	35–60	0.078
IFN-α2b treated	35 (*n* = 21)	25–45	15 (*n* = 21)	5–35	0.011 *
*p* non-treated vs. treated	0.197		0.017 *		–

* Differences are statistically significant (*p* < 0.05).

**Table 3:** Analysis of the level of SpO2 before and after IFN-α2b treatment.

IFN-α2b Treatment	Variables	Follow-Up Periods				*p* Initial vs. Final
		Initial SpO2		Final SpO2		
		Abs.	%	Abs.	%	
IFN-α2b non-treated	normal	23	46.9	34	69.4	0.193
	low	24	49.0	10	20.4	
	very low	2	4.1	5	10.2	
IFN-α2b treated	normal	20	33.9	44	74.6	<0.001 *
	low	31	52.5	10	16.9	
	very low	8	13.6	5	8.5	
*p* non-treated vs. treated		0.149		0.836		–

* Differences are statistically significant (*p* < 0.05).

**Table 4:** Results of the correlation analysis.

Variables	Correlation Characteristics		
	ρ	Strength of the Association Assessed Using the Chaddock Scale	*p*
%CT injuries—Age	0.407	Moderate	0.032 *
%CT injuries—Days in hospital	0.756	Strong	<0.001 *
2SpO2—Days in hospital	−0.544	Close	<0.001 *
%CT injuries—2SpO2	−0.835	Strong	<0.001 *
2SpO2—Age	−0.402	Moderate	<0.001 *

* Differences are statistically significant (*p* < 0.05); 2SpO2—saturation at discharge.

**Table 5:** Characteristics of the association of predictors with the IFN-α2b treatment.

Predictors	Unadjusted		Adjusted	
	COR; 95% CI	*p*	AOR; 95% CI	*p*
Sex: men	1.000; 0.177–5.635	1.000	1.208; 0.054–26.924	0.905
Age	0.993; 0.929–1.061	0.827	1.006; 0.889–1.139	0.923
Days in hospital	0.934; 0.870–1.002	0.057	0.782; 0.534–1.145	0.205
% CT injuries	0.950; 0.908–0.992	0.022 *	0.828; 0.687–0.998	0.047 *
2SpO2	1.083; 0.946–1.241	0.251	0.381; 0.132–1.100	0.074

* Association of the outcome value with the predictor value is statistically significant (*p* < 0.05).

## Data Availability

All the raw data can be provided by the authors upon request.
